# A current perspective on the role of AGCVIII kinases in PIN-mediated apical hook development

**DOI:** 10.3389/fpls.2015.00767

**Published:** 2015-10-06

**Authors:** Björn C. Willige, Joanne Chory

**Affiliations:** ^1^Salk Institute for Biological Studies, La Jolla, CA, USA; ^2^Howard Hughes Medical Institute, La Jolla, USA

**Keywords:** apical hook development, auxin transport, AGCVIII kinases, PIN activity, PIN polarity

## Abstract

Despite their sessile lifestyle, seed plants are able to utilize differential growth rates to move their organs in response to their environment. Asymmetrical growth is the cause for the formation and maintenance of the apical hook—a structure of dicotyledonous plants shaped by the bended hypocotyl that eases the penetration through the covering soil. As predicted by the Cholodny–Went theory, the cause for differential growth is the unequal distribution of the phytohormone auxin. The PIN-FORMED proteins transport auxin from cell-to-cell and control the distribution of auxin in the plant. Their localization and activity are regulated by two subfamilies of AGCVIII protein kinases: the D6 PROTEIN KINASEs as well as PINOID and its two closely related WAG kinases. This mini-review focuses on the regulatory mechanism of these AGCVIII kinases as well as their role in apical hook development of *Arabidopsis thaliana*.

## Introduction

Despite their sessile life-style, land plants are capable of executing a broad range of movements, including extremely fast actions based on the release of stored elastic energy (Edwards et al., 2005) and movements based on the differential growth of plant organs (Gilroy, 2008). In the latter case, one side of an organ elongates faster than the opposing side, leading to organ bending. One example for such a plant movement is the apical hook formation. In etiolated dicotyledonous seedlings, the reduced growth of one side of the hypocotyl leads to the formation of a bended structure, which eases the shoot’s penetration through the covering soil and simultaneously protects its meristem, which will give rise to all postembryonic above-ground organs. The importance of the apical hook for soil penetration was demonstrated by analyses of *Arabidopsis* mutants exhibiting defects in hook development (Harpham et al., 1991).

As predicted by the broadly accepted Cholodny–Went theory, the differential elongation inducing plant movements is dependent on the phytohormone auxin (e.g., indole-3-acetic acid) and its unequal distribution (Cholodny, 1927; Went, 1928). Auxin movement is facilitated by members of three different auxin transporter families (Zazímalová et al., 2010). One of these families is formed by the PIN-FORMED (PIN) auxin efflux transporters (Paponov et al., 2005). Auxin streams and asymmetric growth are regulated by AGCVIII kinases that are able to phosphorylate PINs (Barbosa and Schwechheimer, 2014). The present mini-review summarizes recent research in *Arabidopsis thaliana* pointing out the regulatory mechanisms of two AGCVIII sub-families and the current knowledge of their involvement in the apical hook development.

## Auxin and its Transport by PINs

The phytohormone auxin is involved in virtually all processes of plant growth and development of seed plants. It is mainly synthesized in young leaves as well as the shoot and root apices (Ljung et al., 2001, 2005). The following model describes how the perceived auxin is transferred into transcriptional responses: The soluble receptor TRANSPORT INHIBITOR RESPONSE 1 (TIR1) and its paralogous AUXIN SIGNALING F-BOX (AFB) proteins bind in combination with AUXIN/INDOLE-3-ACETIC ACIDs (Aux/IAAs) auxin and subsequently initiate Aux/IAA degradation by the ubiquitin–proteasome system. Since Aux/IAAs inhibit AUXIN RESPONSE FACTORs (ARFs) by forming heterodimers, their degradation releases the ARFs and allows them to initiate or repress transcription (Mockaitis and Estelle, 2008).

Depending on tissue sensitivity and concentration, auxin can stimulate or inhibit cell growth (Thimann, 1939). This might be explained by the finding that different TIR1/AFB-Aux/IAA combinations have varying auxin affinities. Therefore, growth inhibiting Aux/IAAs might be less stable than growth promoting family members (Calderón Villalobos et al., 2012).

Auxin transport is essential for forming local auxin gradients, maxima and minima and is the consequence of the activity of diverse transporter systems. In the weakly acidic apoplast, a portion of indole-3-acetic acid is protonated which in consequence can pass the apolar cell membrane by diffusion. Nevertheless, the larger proportion of the extracellular indole-3-acetic acid occurs as anion and its import is facilitated by AUXIN RESISTANT1 (AUX1) and LIKE AUXIN RESISTANT1 (LAX) proteins (Marchant et al., 1999; Kramer and Bennett, 2006; Péret et al., 2012). Inside the cells, indole-3-acetic acid is deprotonated and its export is mediated by PIN proteins. PIN1, 2, 3, 4, and 7 are present in the cell membrane and are involved in cell-to-cell auxin transport. The N- and C-termini of PINs consist of clusters of transmembrane domains that are divided by a central soluble domain. This hydrophilic loop is phosphorylated by AGCVIII kinases regulating PIN localization and activity (Vanneste and Friml, 2009; Zazímalová et al., 2010; Barbosa and Schwechheimer, 2014).

## AGCVIII Kinases Phosphorylate PINs

AGC kinases are named after the cAMP-dependent protein kinase A (PKA), cGMP-dependent protein kinase G (PKG) and phospholipid-dependent protein kinase C (PKC) described in animals and yeasts. The genome of *Arabidopsis* encodes for 37 AGC kinases and 23 of them form the plant specific AGCVIII group distinguished from other AGC kinases by a varying insertion in the kinase domain and a conserved and functional mutation in the Mg^2+^ chelating motif necessary for ATP binding (Bögre et al., 2003; Galván-Ampudia and Offringa, 2007; Rademacher and Offringa, 2012).

Two groups of AGCVIII kinases were shown to phosphorylate PIN proteins: PINOID (PID) and the two closely related kinases WAG1 and WAG2 are members of the PID sub-family, while the functionally redundant D6 PROTEIN KINASEs (D6PK and D6 PROTEIN KINASE LIKE1 to 3) form the other group (Michniewicz et al., 2007; Zourelidou et al., 2009; Dhonukshe et al., 2010). The involvement of both sub-families in auxin-dependent processes was suggested by knock-out mutations that cause developmental defects. For example, the shoot apical meristem of *pid* mutants is impaired in the formation of reproductive organs and therefore give rise to naked pin-shaped shoot apices. Defects in embryogenesis can be observed in mutants of both families, while impaired lateral root formation is characteristic for knock-outs of *D6PK* sub-family members. All of these phenotypic characteristics can also be observed in *pin* mutants, pointing to a functional relationship between PIN facilitated auxin transport and AGCVIII kinases (Okada et al., 1991; Bennett et al., 1995; Benková et al., 2003; Zourelidou et al., 2009).

## PIN Polarity is Regulated by PID and WAG Kinases

Depending on tissue and cell type, PIN auxin efflux carriers are polarly localized in the cell membrane, potentially allowing them to dictate the direction of auxin fluxes. For example, PIN1 is basally (the root apex facing) localized in the parenchyma tissue of stems and roots and is involved in the downward transport of auxin (Gälweiler et al., 1998; Friml et al., 2002; Benková et al., 2003).

**FIGURE 1:**
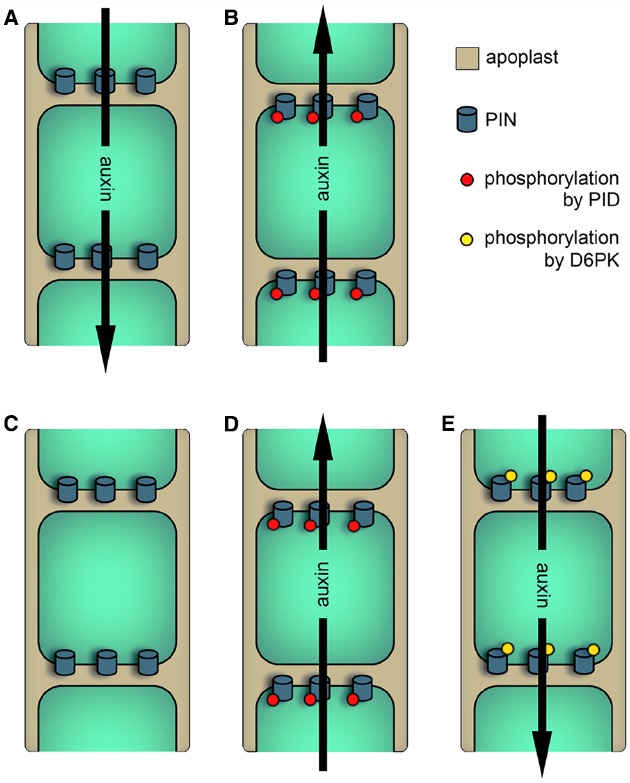
**Models describing the effects of AGCVIII kinase activity on PIN-dependent auxin efflux.** Black arrows represent the direction of auxin streams. **(A)** and **(B)** Older model of PID function. **(A)** In cells with low PID activity (e.g., in *pid* loss-of-function mutants) or in cells expressing PINs with Ser to Ala mutations in the TPRXS(N/S) motifs, PINs are basally localized and therefore facilitate the downward transport of auxin. **(B)** In cells with high PID activity (e.g., in *PID* overexpressors) or in cells expressing PINs mimicking phosphorylation of the TPRXS(N/S) motifs, PIN polarity and hence the direction of auxin transport is changed. **(C)** to **(E)** Newer models of PID and D6PK functions. **(C)** Unphosphorylated PINs are inactive and do not facilitate auxin efflux. **(D)** PID kinase activity modifies PIN localization as well as PIN activity. **(E)** PIN phosphorylation by D6PK activates PINs without regulating PIN polarity.

*PID* overexpressors and mutants argue for a model that describes PID as a regulator of PIN localization (Figures 1A,B): Root cells that show a basal localization of PINs in the wild-type possess an accumulation of apically localized PINs in *PID* overexpressors. Furthermore, the apically located PIN1 protein of the shoot apex is basally localized in *pid* mutants (Friml et al., 2004).

In order to understand the relationship between PID and PINs, *in vitro* phosphorylation studies were performed. Here, it was found that PID phosphorylates the conserved serine residue of three TPRXS(N/S) motifs in the central hydrophilic loop of PIN1. This motif is highly conserved between PIN1 to 4 and 7. Mimicking phosphorylation of all three sites in PIN1 leads to a, although not complete, basal to apical polarity shift in root cells. Additionally, serine to alanine mutations in the phospho-motifs replicate the apical to basal shift of PIN1 observed in shoot apices of *pid* mutants (Huang et al., 2010). These observations indicate a link between PIN phosphorylation by PID, PIN localization and the potential direction of auxin transport.

Overexpression and *in vitro* phosphorylation studies analyzing WAG1 and 2 indicate that both kinases utilize the same or a similar mechanism like PID to control PIN polarity. Conversely, the overexpression of *D6PK*s does not influence PIN polarity. This suggests that these proteins utilize are different mechanism to modify auxin transport (Zourelidou et al., 2009; Dhonukshe et al., 2010).

## PIN Efflux Activity is Triggered by D6PK and PID Family Members

Mutations of *D6PK*s can lead to strongly impaired basipetal auxin transport, indicating a role of the kinases in stimulating auxin streams. Similar to PID, D6PK is also capable of phosphorylating the hydrophilic loop of PIN proteins, but both kinases show a differential preference regarding their targeted phospho-sites: the TPRXS(N/S) motifs are weaker phosphorylated by D6PK and phosphorylation mainly takes place at two additional sites. Conversely, the latter two sites are only poorly phosphorylated by PID. Both sites favored by D6PK are conserved in PIN3, 4, and 7, while only one of the two sites is preserved in PIN1 (Zourelidou et al., 2009, 2014).

To test the role of D6PK, PID, and WAG2 in regulating auxin transport activity, *Xenopus* oocytes were used as a heterologous expression system. Here, PIN1, or PIN3 alone were unable to enhance auxin efflux, whereas their co-expression with one of the three AGCVIII kinases stimulates the outward transport of auxin. This activation seems to be completely dependent on PIN phosphorylation, as mutating the phospho-sites blocked the activation of auxin transport by the AGCVIII kinases (Zourelidou et al., 2014).

In summary, both sub-families seem to have an overlapping but distinct function in regulating auxin transport. This is also supported by promoter swap experiments showing that PID and D6PK are not functionally replaceable (Zourelidou et al., 2014). PID and the WAGs modulate PIN localization and activity and both events are seemingly not separable, because phosphorylation of the TPRXS(N/S) motifs control both properties (Dhonukshe et al., 2010; Huang et al., 2010; Zourelidou et al., 2014; Figures 1C,D). Unlike PID, D6PK does not modify PIN polarity and shows an exclusively basal localization (Zourelidou et al., 2009; Dhonukshe et al., 2010; Barbosa et al., 2014). This indicates that D6PK controls the strength of the PIN-dependent downward auxin transport, which is the proposed major polar auxin route in the plant body (Figures 1C,E).

## Potential PIN-dependent Auxin Routes Mediating Apical Hook Development

The development of the apical hook can be divided into three phases: formation, maintenance and opening. During the formation phase, the apical hypocotyl bends downwards till the hook forms an angle of about 180°. The apical hook retains this angle during the subsequent maintenance phase. These two phases take about 24 h each. Eventually, the hook opens gradually. This takes several days in the dark, but only a couple of hours after light exposure (Liscum and Hangarter, 1993; Raz and Ecker, 1999; Vandenbussche et al., 2010; Zádníková et al., 2010).

The hook’s bending and its maintenance are mainly based on unequal cell elongation between the inner (concave) and outer (convex) side (Silk and Erickson, 1978; Raz and Ecker, 1999). Additionally, the convex side consists of slightly more cells than the concave side (Raz and Koornneef, 2001). The supposed prerequisite for the inhibited cell elongation in the inner side is the formation and maintenance of a local auxin maximum, which was demonstrated by auxin measurements and by the visualization of *DR5* auxin signaling reporters (Schwark and Schierle, 1992; Friml et al., 2002; De Grauwe et al., 2005). Several tissues have been proposed to be the source of auxin for this maximum: auxin is produced in the cotyledons, but biosynthesis might also occur in the shoot apical meristem and the apical hook region itself (Ljung et al., 2001; Stepanova et al., 2008; Vandenbussche et al., 2010). Nevertheless, studies using auxin transport inhibitors and auxin transport mutants clearly demonstrate that the formation of the apical hook and the repression of its opening are dependent on auxin transport. At least PIN1, 3, 4, and 7 are involved in apical hook formation and maintenance, with PIN3 having a predominant role (Lehman et al., 1996; Friml et al., 2002; Vandenbussche et al., 2010; Zádníková et al., 2010).

How these PINs direct the auxin flow to generate the auxin maximum in the concave side of the hook and how the auxin streams change to induce the opening phase is not fully understood. This is partly based on the attenuation of fluorescent signal intensities in whole-mount apical hooks using confocal microscopy. It is only in the hook, but not in the more basal hypocotyl, that fluorescence signals of tagged PIN proteins are merely visible in the outer cell files of confocal sections. For example, PIN3 promoter GUS analyses as well as cross sections and epifluorescence studies of natively expressed PIN3:GFP clearly demonstrate its expression in the stele and the endodermis of the apical hook (Zádníková et al., 2010; Gallego-Bartolomé et al., 2011; Willige et al., 2012). In contrast, longitudinal confocal sections of the same region only show fluorescence signals in the epidermis and the cortex cells (Zádníková et al., 2010; Willige et al., 2012; Boutté et al., 2013). The cause for this effect is probably the opaqueness of the hook region leading to scattering of the incoming laser light.

Nevertheless, the following mechanism was proposed based on the observation of higher PIN levels in the epidermis and cortex of the convex side: In the hook, auxin transported in the stele is transferred laterally through the endodermis to the outer cell files. Here, the increased PIN levels on the convex side raise the draining of auxin from the outer side out of the hook and hence establish an auxin gradient between both sides (Zádníková et al., 2010, Figure 2A).

**FIGURE 2:**
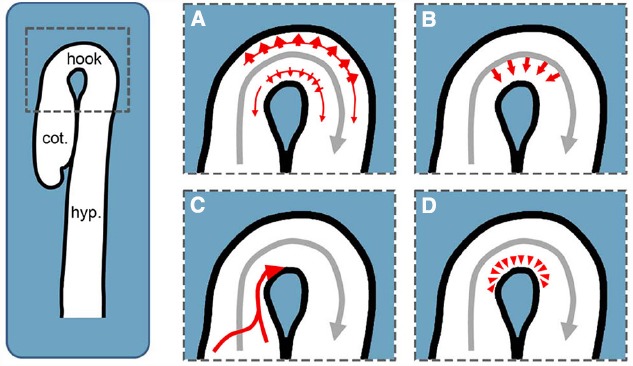
**Potential PIN-dependent auxin transport routes for establishing the apical hook’s auxin maximum.** Gray arrows represent basipetal auxin transport in the stele, whereas red arrows illustrate the potential auxin routes establishing and maintaining the maximum in the apical hook. **(A)** Model proposed by Zádníková et al. (2010): Higher PIN abundance in the cortex and epidermis of the convex side of the hook enhances the draining of auxin to establish an auxin gradient between both sides. **(B)** Differential PIN abundance, activity or localization might lead to a preferential auxin transport from the stele through the endodermis into the outer cell files of the concave side. **(C)** Independent of the basipetal auxin transport in the stele, auxin might be transported through the epidermis from the cotyledons into the concave side of the apical hook. **(D)** In addition to the other potential auxin routes, polar transport might trap the hormone in order to maintain a local maximum. cot.: cotyledons, hook: apical hook, hyp.: hypocotyl.

Alternatively, but not necessarily in conflict with this proposed model, the formation of the auxin maximum could be the consequence of auxin fluxes specifically targeting the concave side to selectively raise the local auxin concentration. This is a strong possibility because the auxin located in the apical hook supposedly reaches a concentration surpassing growth promoting levels. To achieve this, following transport routes are conceivable: PIN3 in the endodermis may preferentially direct the auxin transported in the stele into the inner side of the hook (Figure 2B). This mechanism could be based on PIN3 polarity and would resemble the proposed mechanism for gravi- and phototropic hypocotyl bending (Ding et al., 2011; Rakusová et al., 2011). Otherwise, it could be based on higher PIN3 activity or protein levels in the endodermal cells facing the inner side. Additionally, auxin fluxes along the outer cell layers are proposed transport routes during hypocotyl phototropism and root gravitropism (Ottenschläger et al., 2003; Abas et al., 2006; Christie et al., 2011). Having this in mind, a basipetal auxin flux along the outer cells of the cotyledons and the cotyledon petioles and eventually into the epidermis of the inner side of the apical hook may form a conceivable auxin transport route (Figure 2C). Furthermore, PINs in the cells forming and surrounding the concave side of the hook might trap auxin by pumping the escaping hormone back into this region (Figure 2D).

## Apical Hook Development is Dependent on AGCVIII Kinases

Currently, there is no study available focusing on the role of D6PKs in apical hook development. Nevertheless, 3 days old etiolated *d6pk d6pkl1* double and *d6pk d6pkl1 d6pkl2* triple mutants exhibit a completely opened hook. This indicates a necessity of D6PK activity for the formation or maintenance of the apical hook. This is supported by *DR5* reporter analyses: Instead of the auxin signaling maximum in the concave side of the apical hook evident in wild-type seedlings, *d6pk* triple mutants possess an increased signal in the cotyledons, but no *DR5* activity in the potential hook region (Willige et al., 2013; Barbosa et al., 2014). The open apical hook and the intensified *DR5* expression in the cotyledons strongly resemble the effect of auxin transport inhibitors (Lehman et al., 1996; Vandenbussche et al., 2010; Willige et al., 2012). This indicates that these inhibitors and the reduced D6PK action impair the auxin flux out of the cotyledons, which supposedly serves as auxin source for the apical hook.

The *PID* sub-family member *WAG2* is expressed in the cotyledons and the inner side of the apical hook. While the apical hook formation is not affected in *wag2*, hook opening appears faster in the mutant, indicating that WAG2 represses apical hook opening. This role of WAG2 later in hook development is further reflected by a strong reduction of *DR5* activity in the concave side of the hook. This difference is much less pronounced earlier in hook development. Interestingly, also the cotyledons show a strong reduction of the *DR5* signal, which is restricted to the cotyledon tips and overlaps with a local expression maximum of *WAG2* (Willige et al., 2012). Based on all these observations, WAG2 might be involved in the following functions by regulating PIN polarity and/or activity: In the cotyledons, the kinase may participate in transmitting auxin from the tip into the direction of the cotyledon base and the hypocotyl, while WAG2 action in the concave side of the hook is probably involved in maintaining a high local auxin concentration.

In contrast to *wag2* mutants, loss of *WAG1* function does not interfere with hook development (Willige et al., 2012). Nevertheless, *pid* quadruple mutants (carrying mutations in *PID*, its closest homolog *PID2* and the two *WAG*s) have a slightly opened hook early in development (Haga et al., 2014). This may indicate a redundant function of *PID* sub-family members in apical hook formation, or might be the consequence of the quadruple mutant’s defective embryogenesis: these mutants lack cotyledons and likely produce less auxin for the apical hook formation (Cheng et al., 2008; Dhonukshe et al., 2010).

## Regulation of *WAG2* Expression During Apical Hook Development

*WAG2* is transcriptionally activated by gibberellins, a class of phytohormones indispensable for the formation and maintenance of the apical hook (Achard et al., 2003; Vriezen et al., 2004; Gallego-Bartolomé et al., 2011; Willige et al., 2012). Additionally, *WAG2* expression diminishes after exposure to light. Interestingly, a block of gibberellin signaling or exposure to light disrupts the hook’s auxin maximum in the concave side (Wu et al., 2010; Gallego-Bartolomé et al., 2011; Willige et al., 2012).

PHYTOCHROME INTERACTING FACTORs (PIFs) are integrators of gibberellin and light signaling, since their activity is repressed by DELLA proteins (the negative regulators of gibberellin signaling) and their degradation is induced after exposure to light (Schwechheimer and Willige, 2009). Multiple *pif* mutants show a de-etiolated phenotype in the dark, including a strongly impaired apical hook development (Leivar et al., 2008). Furthermore, the loss of PIFs leads to a reduced *WAG2* expression. PIF5 plays a major role in the transcriptional activation of *WAG2* and was shown to directly bind to its promoter (Willige et al., 2012). Altogether, these data suggest following model for a *WAG2*-dependent hook opening after the seedling’s penetration of the covering soil: The perceived light induces PIF degradation and lowers gibberellin levels (Ait-Ali et al., 1999; Achard et al., 2007; Alabadí et al., 2008), which then leads to a repression of *WAG2* promoter activity. This decrease in WAG2 abundance lowers the PIN-dependent auxin streams that are necessary for maintaining the hook’s auxin maximum. As a result, auxin levels drop below a growth inhibiting concentration and induce cell elongation in the concave side.

## Perspective

The finding that AGCVIII kinases do not only regulate PIN localization, but also transporter activity, indicates that knowledge of PIN polarity is insufficient to predict auxin streams. Nevertheless, a complete picture about auxin transporter distribution is necessary to understand the auxin fluxes leading to the maximum in the concave side of the apical hook. These studies should not be limited to the hook region, since it is necessary to include auxin producing organs such as the cotyledons. As described above for the apical hook, analyzing fluorescent tagged proteins in deeper tissues of whole-mount cotyledons using confocal microscopy is challenging, due to low light transmission. This problem might be circumvented by analyzing cross-sections, but the use of perfluorocarbons (PFCs) allows to image intact leaves by diminishing the issue of attenuated excitation laser intensity (Littlejohn et al., 2010, 2014). PFCs might also improve the microscopy in the apical hook region itself. Alternatively, clearing techniques to enhance transmission of light through the cotyledons and the apical hook should help to improve our understanding of auxin transporter polarity in these opaque tissues (Warner et al., 2014). These studies should not be restricted to PINs since it was demonstrated that the auxin transporters AUX1, LAX3 as well as ATP-BINDING CASSETTE B1 and B19 are also involved in apical hook development (Vandenbussche et al., 2010; Wu et al., 2010).

Furthermore, the use of phospho-site specific PIN antibodies for immunostainings (Marhavý et al., 2014; Zourelidou et al., 2014) might give us an idea about the PIN activity in the cells forming the apical hook. Altogether, the understanding of PIN polarity and activity with complementary studies of the other auxin transporter families should deliver a comprehensive understanding of auxin fluxes during apical hook formation, maintenance and opening. Additionally, the analyses of uncharacterized AGCVIII kinases might lead to the identification of important auxin transport regulators during hook development.

### Conflict of Interest Statement

The authors declare that the research was conducted in the absence of any commercial or financial relationships that could be construed as a potential conflict of interest.
